# Impact of Brightness on Choroidal Vascularity Index

**DOI:** 10.3390/jcm13041020

**Published:** 2024-02-10

**Authors:** Nicola Rosa, Marco Gioia, Rachele Orlando, Martina De Luca, Eleonora D’Aniello, Isabella Fioretto, Ciro Sannino, Maddalena De Bernardo

**Affiliations:** Eye Unit, Department of Medicine, Surgery, and Dentistry “Scuola Medica Salernitana”, University of Salerno, via S. Allende, 84081 Baronissi, Salerno, Italy; nrosa@unisa.it (N.R.); marco.gioia@yahoo.it (M.G.); eldaniello@unisa.it (E.D.); isa.fioretto@libero.it (I.F.); cirosanninodr@gmail.com (C.S.); mdebernardo@unisa.it (M.D.B.)

**Keywords:** blooming effect, choroid, CVI, EDI-OCT, OCT

## Abstract

The use of choroidal vascularization to diagnose and follow-up ocular and systemic pathologies has been consolidated in recent research. Unfortunately, the choroidal parameters can be different depending on the lighting settings of optical coherence tomography (OCT) images. The purpose of this study was to examine whether the brightness of OCT images could influence the measurements of choroidal parameters obtained by processing and analyzing scientific images with the ImageJ program. In this observational, prospective, non-randomized study, 148 eyes of 74 patients with a mean age of 30.7 ± 8.5 years (ranging from 23 to 61 years) were assessed. All patients underwent a complete ophthalmological examination including slit lamp, fundus oculi, ocular biometry, corneal tomography and spectral domain (SD) OCT evaluations of the foveal region in the enhanced depth imaging (EDI) mode. OCT images at two different brightness levels were obtained. The total choroidal area (TCA), choroidal vascularity index (CVI), stromal choroidal area (SCA) and luminal choroidal area (LCA) at both lower and higher brightness levels were measured. To avoid the bias of operator-dependent error, the lower and higher brightness TCAs were obtained using two methods: the manual tracking mode and fixed area. At the two different brightness levels, LCA, SCA and CVI measurements showed statistically significant changes (*p* < 0.05), whereas the TCA differences were not statistically significant (*p* > 0.05). According to the results of this study, highlighting that brightness could affect LCA, SCA and CVI parameters, care should be taken during OCT image acquisition.

## 1. Introduction

The choroid, the posterior uveal tract, is mainly composed of blood vessels, connective tissue, melanocytes, nerve fibers and extracellular fluid [1]. It can be differentiated into three layers: the choriocapillaris, the Sattler and the Haller layers, with increasing vessel diameters from the inside to the outside [2]. In the last few years, several authors have suggested a possible relationship between choroidal changes and degenerative or inflammatory ocular diseases, such as polypoidal choroidal vasculopathy (PCV) [3,4,5], exudative and non-exudative age-related macular degeneration (AMD) [6,7,8,9,10], high myopia [11,12,13] and intermediate uveitis [14,15,16], respectively, and the treatment of these illnesses [16,17,18,19,20,21,22]. The possible relationship between choroid and respiratory or cardiovascular disorders, such as obstructive sleep apnea syndrome [23,24] and atherosclerosis requiring carotid stenting [25,26], was also studied in depth.

However, in science, and even more in medicine, doubting the reliability of what is measured is very important.

Among the tools utilized to investigate the choroidal layers, image J software is one of the most used. This software allows us to measure the total choroidal area (TCA), choroidal vascularity index (CVI), stromal choroidal area (SCA) and luminal choroidal area (LCA), but, so far, we have not been able to find papers discussing the reliability of these kind of measurements and the possible bias that could affect these choroidal parameters [2].

Recently, some authors have suggested that these choroidal parameters could be influenced by the brightness of the images [11,14,23,25,27], but to the best of our knowledge no papers have been published on this issue. Hence, the purpose of this study is to evaluate whether different values of brightness in enhanced depth imaging (EDI) optical coherence tomography (OCT) scans could influence the CVI, TCA, LCA and SCA choroidal parameters.

## 2. Materials and Methods

### 2.1. Selection of Patients

In this study, healthy subjects were recruited between September 2020 and May 2021. Patients with systemic and ocular diseases, or who had had previous eye surgery, were aged under 18 or over 70 years old, had an axial length (AL) < 21 mm and >27.5 mm, and pregnant women were excluded. All participants were thoroughly informed about the purpose of the study and informed written consent was obtained. Institutional Review Board (IRB) approval was also obtained from the ComEtico Campania Sud (CECS), prot. n°16544.

### 2.2. Clinical and Instrumental Examination

All patients underwent a complete eye examination. The evaluation included AL measurement with an IOLMaster (Carl Zeiss Meditec AG, Jena, Germany, version 5.4.4.0006) and spectral domain OCT evaluation in an EDI mode (Spectralis; Heidelberg Engineering; Heidelberg, Germany, version 6.0).

### 2.3. OCT Analysis

The OCT analysis was performed using the included OCT software (1.0) measurement tool. The macular region was evaluated through a horizontal 30° linear OCT B-scan passing through the fovea, with an average of 100 frames for every B-scan [2]. All subjects were analyzed between 2:00 p.m. and 4:00 p.m., to reduce bias related to diurnal variations of choroidal thickness (ChT), and then CVI measurements were performed by an expert examiner using the software ImageJ 1.52 [2]. As described by Agrawal et al.’s protocol, all macular scans were first binarized using Niblack’s autolocal threshold tool. This method resulted in their conversion from a grayscale image into a black and white image, a binary scale, and resulted in a clear observation of the choroid scleral junction [2]. The “polygon” tool was applied and a choroidal area bounded 1500 microns nasally and temporally from the fovea, whose upper limit was traced along the margin of the retinal pigmented epithelium (RPE)and lower limit along the choroid–scleral interface, was selected and added to the ROI Manager of ImageJ, in the region of interest section. The total tracked area corresponds to the TCA. To select the dark pixels to obtain the LCA, the image was converted to red, green, blue (RGB). SCA was calculated by subtracting the LCA from the TCA. CVI was calculated as the ratio between the LCA and TCA [2].

In the present study, two TCA tracking modes were used:

- A classic manual tracking method, manually drawing the lines to limit the choroidal area for each high and low brightness level [2].
- An alternative method, with a fixed area selection independent of the brightness level.

**Figure 1:**
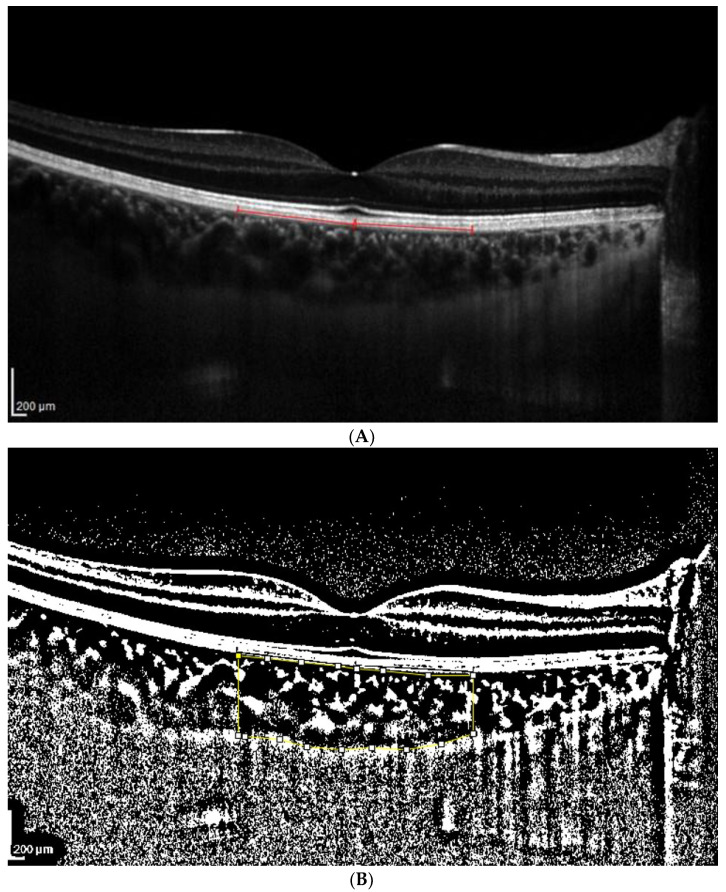

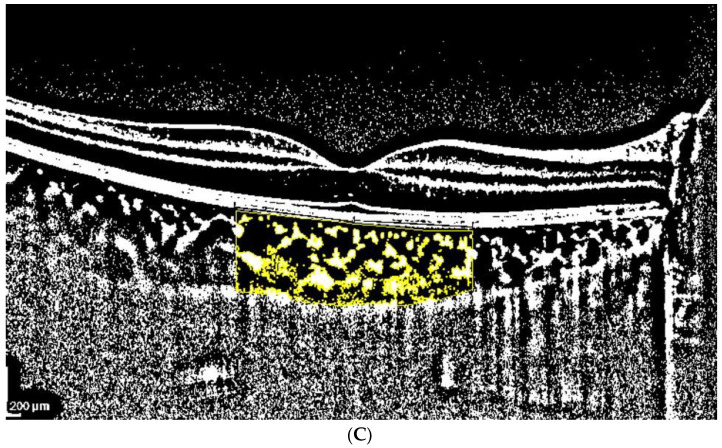
(**A**). Patient 1 EDI-OCT right eye scan at 8 level brightness. Red line indicates the RPE. (**B**). Patient 1 EDI-OCT binarized right eye scan at brightness 8. Assessment of total choroidal area. (**C**). Patient 1 EDI-OCT binary right eye scan at 8 brightness. Assessment of luminal choroidal area and stromal choroidal area. Yellow lines highlight the yellow choroidal area being calculated.

**Figure 2:**
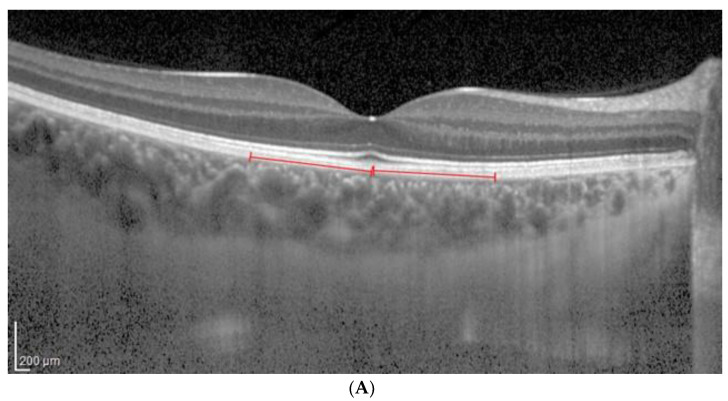

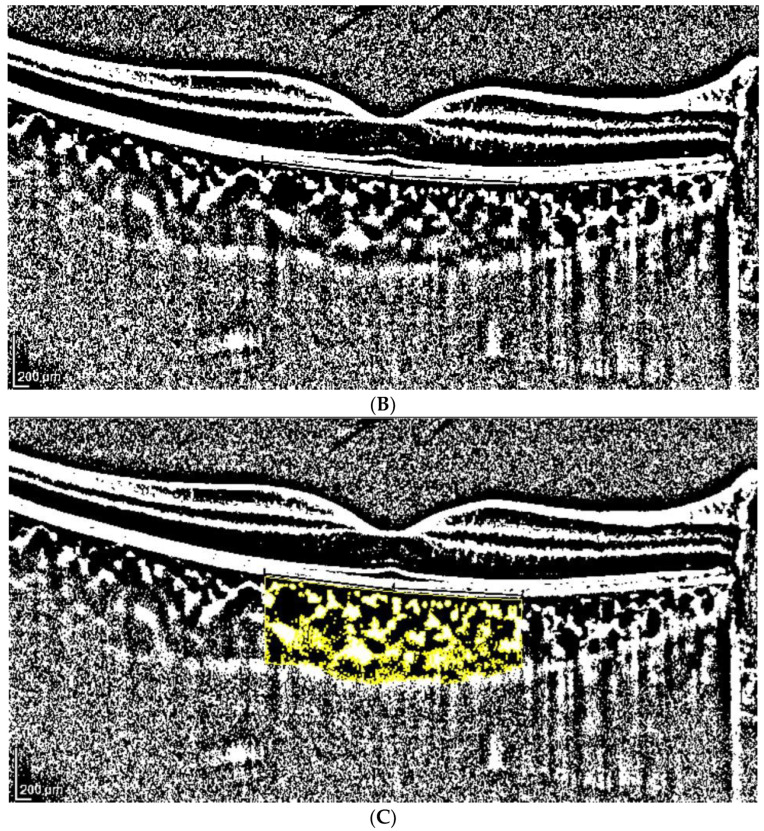
(**A**). Patient 1 EDI-OCT right eye scan at 16 brightness. Red line indicates the RPE. (**B**). Patient 1 EDI-OCT right eye scan at brightness 16. Assessment of total choroidal area. (**C**). Patient 1 EDI-OCT right eye scan at 16 brightness. Assessment of luminal choroidal area and stromal choroidal Area. Yellow lines highlight the yellow choroidal area being calculated.

The CVI, LCA, SCA and TCA were calculated, taking into account the two modes above for each acquisition and utilizing brightness levels 8 and 16, as shown in Figure 1 and Figure 2.

### 2.4. Statistical Analysis

Data collection was performed using Microsoft Excel, and statistical analysis using IBM SPSS Statistics Version 26 (International Business Machine Corporation, Armonk, NY, USA). Kolmogorov–Smirnov Test was performed to assess normal distribution of the data. Wilcoxon test was applied to non-normally distributed data, whereas a paired T test was applied to normally distributed data.

In addition, the mean, standard deviation, median, minimum and maximum were calculated in each group and for each parameter. Results were considered statistically significant when *p* < 0.05.

The sample size was determined by maximizing the statistical power. The analysis was performed using G*Power software (version 3.1.9.4).

The sample size was calculated for the paired T test as follows: α error = 0.05, 1-β error = 0.95 and effect size = 0.299 were set and a noncentral parameter δ = 3.64, critical t = 1.98, Df = 147, total sample size = 148 and an actual power = 0.951 were obtained. Moreover, the sample size for the Wilcoxon test was assessed: α error = 0.05, 1-β error = 0.95 and effect size = 0.279 were set and a noncentral parameter δ = 3.32, critical t = 1.65, Df = 54.38, total sample size = 148 and actual power = 0.951 were obtained.

## 3. Results

In total, 148 eyes of 74 patients (34 males and 40 females), with a mean age of 30.7 ± 8.5 years (ranging from 23 to 61 years) and an AL of 24.2 ± 1.1 mm (ranging from 21.6 to 27.3 mm), were evaluated.

The results obtained using the TCA manual tracking mode are shown in Table 1 and in Figure S1, Figure S3, Figure S5 and Figure S7 in the Supplementary Materials, while the ones obtained using the TCA fixed tracking mode are shown in Table 2 and in Figure S2, Figure S4, Figure S6 and Figure S8 in the Supplementary Materials. Utilizing both tracking methods, the measurements obtained for the LCA, SCA and CVI with the two different brightness levels showed statistically significant changes (*p* < 0.05), and these are summarized in Figures S1–S6 in the Supplementary Materials and Table 1 and Table 2, whereas no statistically significant changes for the TCA measurements were detected (*p* > 0.05), and this is recorded in Figures S7 and S8 in the Supplementary Materials and Table 1 and Table 2.

## 4. Discussion

The choroidal stroma and vessels were first investigated using histological studies. [28] These later were able to demonstrate the significant choroidal structural changes in several diseases, although the results were affected by vascular size alterations during the tissue fixation processes. Fryczkowski AW et al. studied 24 autopsy eyes from patients with long-standing type 1 Diabetes Mellitus using scanning electron microscopy, and compared them to 10 autopsy eyes from healthy subjects, and showed significant alterations of the uveal tract in the first group’s eyes, such as increasing tortuosity, dilation and a narrowing of the choroidal vessels [28].

In 2008, Spaide et al. [29] proposed an EDI-OCT system. They obtained an inverted representation of the choroid using an SD OCT device that was close enough to the eye of healthy volunteers without causing pupillary dilation. This was an innovative method to better visualize and measure ChT in vivo. Then, Tian et al. presented a fast and accurate algorithm to segment and quantify the choroid either manually or through specific algorithms [1]. Analyzing high-contrast choroidal cross-sectional images, the complex of pixels with the biggest gradient value above the RPE was identified as Bruch’s membrane; then, using Dijkstra’s algorithm, the shortest path on the graph formed by the valley pixels was detected and identified as the choroidal–scleral interface [1].

Initially, ChT was thought to be used as an indicator of both systemic and ocular individual health status, because changes in this parameter have been observed in many pathological disorders [30,31,32,33,34]. Lindner et al. found a thinner choroid in 72 eyes of 72 patients with geographic atrophy (GA) compared to 37 eyes of 37 healthy patients and a correlation between ChT and the GA subtype, showing that ChT reflects the disease’s heterogeneity [30]. Young et al. showed a progressive choroidal thinning in 22 eyes of 11 patients with clinically inactive uveitis, such as birdshot chorioretinopathy [31]. Kim et al. studied 235 eyes of 145 patients, 40 of whom underwent a laser panretinal photocoagulation (PRP), while 195 were treatment-naïve and categorized based on the presence and severity of diabetic retinopathy. They demonstrated a direct proportionality between ChT and the severity of diabetic retinopathy; moreover, they demonstrated a thicker subfoveal choroid in patients with diabetic macular edema and the thickest ChT in edemas with subretinal detachment [32]. De Bernardo et al. studied 25 eyes of 25 patients undergoing alpha-lytic therapy and 25 eyes of 25 healthy controls, both programmed for cataract surgery in the fellow eye. They found a decrease in ChT and no floppy iris syndrome during surgery in patients after drug withdrawal [33]. De Bernardo et al. explored 64 celiac patients and 67 healthy subjects and found that the subfoveal TCA, LCA, SCA and subfoveal ChT, but not CVI, were significantly different between the two groups [34]. However, further studies showed that ChT cannot be considered a valid marker, because it is too susceptible to biological variables, such as intraocular pressure or AL, which present an inverse correlation with ChT [2,35,36]. Moreover, it is well known that AL measurements are not very precise, and the use of correcting formulas in AL measurements could change this finding [37]. Another limitation of ChT measurements is that no information on vessels or stromal changes can be obtained [36]. To overcome these problems, a new parameter, namely CVI, was subsequently introduced by Agrawal et al. [2]. In their studies, the choroidal image was acquired again using the EDI-OCT mode, in particular, by first scanning the macular region and then binarizing it according to the Sonoda et al. protocol, making minor changes [36]. This results in a black and white image, on which three components can be identified: the TCA, LCA and SCA.

The CVI is calculated as a ratio between the LCA and TCA [2]. Since its introduction, the CVI has been widely used in international research, but recently some concerns have been raised about its reliability, due to the possible influence of the image brightness at acquisition time on its evaluation [11,14,23,25,27] or the influence of dark- or light-adapted eyes and hyperglycemia on the resulting CVI [38]. Although OCT-angiography (OCTA) allows us to study in vivo retinal and choroidal vascularization, similar limitations due to signal attenuation are present even in OCT-angiography (OCTA), as denoted by Di Pippo et al. in their comparison between EDI-OCT and OCTA for evaluating AMD [39].

Chung et al. analyzed 25 eyes with PCV, 14 uninvolved fellow eyes, 30 eyes with exudative AMD, 17 eyes with early AMD and 20 eyes of age-matched normal subjects. They discovered a significant thickening of PCV patients’ choroids compared with AMD patients and deduced a complex pathogenesis of PCV disease. Unfortunately, in the study, several images with different brightnesses and different appearances of the choroid were presented, raising the question of possible bias in evaluating the vascular and stromal components [3].

A similar problem was observed by He M et al. and Wu CY et al. in assessing the CVI in patients with obstructive sleep apnea syndrome [23,24]; Li S et al. and Kim JH et al. in patients after carotid stenting [25,26]; Fujiwara T et al., Wang S et al. and Xiong K et al. in patients with high myopia [11,12,13]; Przybyś M et al., Kim M et al. and Gómez-Gómez A et al. in patients with intermediate uveitis [14,15,16]; and Wei X et al. in smoker patients [27].

Furthermore, although the brightness level is not modified, sometimes a change in the focus or simply a lens tilting during the OCT acquisition process can induce a change in the image brightness.

The possibility of modifying the brightness in some cases can be an advantage; in fact, decreasing the brightness can allow us to better differentiate very high-reflective lesions from medium–high-reflective ones, as in case of optic nerve drusen detection [40]. On the other hand, increasing the brightness can better visualize the presence of low-reflective lesions that could be missed when utilizing a higher brightness. This effect has been known for years in the echographic field, with the name the blooming effect, meaning that low reflective echoes will blow up, increasing the system’s sensitivity and so creating brighter images. Unfortunately, this effect makes the images’ measurement unreliable; in fact, as with high signal amplification, the signals from low reflective structures will blow up, and the black images will appear smaller compared to a lower amplification, as the low reflective signals will disappear and the image will show larger darker areas [41,42,43,44].

In the echographic field, this problem has been overcome by utilizing a standardized A scan, which has a specific “tissue sensitivity” which is obtained by employing a tissue model that is not available for B-scans [45].

The need to utilize an optimal system sensitivity setting to obtain repeatable results has been known about for years. In fact, even in the early 60s of the last century, a variety of reflectors such as glass or steel surfaces in water were used, but the obtained echoes were not comparable to those obtained from tissue, and it was clear that scattering tissue models should have been used. For this reason, a variety of fresh human tissue such as normal liver or formalin-fixated tissues, or suspensions of tumor cells obtained from rat or mouse ascites, were used, but the results were poor. Finally, citrated human blood was shown to be a useful biological standard [45]. However, the need for a simpler and especially more permanent model encouraged investigators to look for an inorganic material which could replace human blood. Finally, in 1975, P. Till succeeded in finding such a material and created a solid tissue model that has been used since then to calibrate A scan probes. It is made of a matrix of silicon resin (Wacker Sil Gel 504) containing a specific number of glass micro-beads (S100) with equal distribution [45].

For this reason, in the echographic field B-scan is mainly used for detecting lesions, but, in the case of taking measurements, the standardized A scan technique is preferred [44].

In our opinion, this problem is also present in cases of OCT measurements, explaining the reason why conflicting results are present in several papers.

In a study by Wei et al. [46], EDI OCT was performed in 100 eyes of 52 patients and images were binarized separately using Sonoda’s [36] and Agrawal’s techniques [2]. Then, the CVI was measured, resulting in a poor agreement between the two binarization techniques (ICC of 0.353).

In addition, in treated Vogt-Koyanagi-Harada (VKH) disease, the estimated CVI was discrepant between different studies.

Liu et al. selected 40 patients with chronic VKH disease with no active inflammation and 40 healthy subjects. EDI-OCT was performed at baseline, during the recurrence of an anterior uveitis attack and 1 week after the anterior uveitis resolved. The authors calculated the CVI according to Agrawal’s protocol, showing a CVI increase after treatment (*p* < 0.0001) [47].

Kawano et al. found a CVI increase after treatment (*p* < 0.01) too. EDI-OCT images from 32 eyes of 16 patients with treatment-naïve VKH were acquired at the baseline, 1 week and 1 month after initiating steroid therapy, and then they were processed according to Sonoda’s protocol [48].

On the contrary, Agrawal et al. collected EDI-OCT images from 18 eyes of 9 patients with VKH at baseline, within 2 weeks of acute presentation, and again at 6 to 12 months. The images were binarized according to Sonoda’s protocol and, after treatment, the CVI was found to be significantly reduced from the baseline (*p* < 0.0001) [49].

Likewise, Jaisankar et al. conducted EDI-OCT examinations in 16 eyes treated with systemic corticosteroids for active VKH. The images were binarized according to Sonoda’s protocol, resulting in a significant reduction in the post-treatment CVI (*p* = 0.02) [50].

Contradictory results have also been shown in Non-Arteritic Anterior Ischemic Optic Neuropathy (NAION).

Pellegrini et al. collected OCT scans from 20 patients with A-AION resulting from temporal arteritis, 20 patients with NA-AION and 20 controls, binarized them as stated by Agrawal R et al. [2], and demonstrated no significant difference in the macular and peripapillary CVIs between NA-AION patients and the controls (respectively, *p* = 0.942 and *p* = 0.570) [51].

On the other hand, Guduru et al. captured OCT images of 20 eyes of 20 patients with acute unilateral NAION and 40 eyes of 40 healthy patients; the authors applied Agrawal’s binarization method and observed a significant difference in the CVIs of the nasal and temporal areas between NAION eyes and healthy patients (both *p* < 0.001) and in the temporal CVI between NAION eyes and their healthy fellow eyes (*p* = 0.007) [52].

Some authors have tried to answer to these criticisms by providing arguments such as:

- Image binarization techniques use special algorithms such as Niblack, which can isolate vascular structures. In reality, there is no certainty that the darker areas of OCT images correspond to the luminal component and the lighter areas to the stromal component, although the literature supporting this hypothesis is robust [53].
- So far, in the literature, no clear evidence exists that the blooming effect could be present in OCT images, considering the different nature of the signal from ultrasound, which uses an ultrasonic signal, while OCT uses interferometry [52].

To check for the eventual presence of the blooming effect in this study, two tracking modes of TCA were used: the classic manual method of tracking and a fixed total area. The first method is more susceptible to operator-dependent errors, while the second is more objective, because it utilizes a fixed total area which remains constant while changing the brightness. In both cases, a statistically significant difference in the LCA, SCA and CVI parameters was found. In particular, when increasing the OCT brightness levels, the number of brighter areas increase, resulting in an increase in the SCA, while darker areas decrease, resulting in a reduction in the LCA. These differences increase with the increase in the size of the evaluated areas (Figures S1–S4). Therefore, in the first method, the CVI measurements will be lower than those obtained in the second method.

## 5. Conclusions

In the literature, the CVI ranges from 40% to 70%. Different brightnesses of OCT images, as well as different and operator-dependent tracking modes could explain the CVI values’ ambiguity. The aforementioned differences represent the limitations of this method for evaluating choroidal vascularization, which has been validated and used in many manuscripts in the most recent literature [2,15,27,35,36,39,46,47,48,49,50,51,52,53].

Diagnostic imaging is the only method used to calculate the CVI, and its histological findings are invalidated by alterations in vascular and stromal structures during the preliminary phases of the tissue’s fixation and study [14]. For this reason, solving this uncertainty is quite difficult.

To overcome these limitations, it is advisable to establish a standardized method when performing CVI evaluations. This should include a standardized OCT software that can always use the same brightness during image acquisition, an automatic choroidal segmentation and an automatic method for drawing a pre-established total area. However, to validate this process further studies are needed.

## Figures and Tables

**Table 1:** TCA, LCA, SCA, CVI and their differences (in %), using the classic (manual) tracking mode for the total choroidal area, at brightness levels 8 and 16. TCA, LCA and SCA are in mm^2^.

**TCA Low vs. High Brightness**				
	**Low**	**High**		
	**TCA**	**TCA**	**Modification (%)**	***p***
Average	3.52	3.54	0.87	<0.681
SD	1.13	1.12	7.47	
Median	3.49	3.50	−0.24	
Min	1.12	1.01	−26.95	
Max	6.94	6.97	30.60	
**LCA Low vs. High Brightness**				
	**Low**	**High**		
	**LCA**	**LCA**	**Modification (%)**	***p***
Average	2.65	2.44	−8.52	<0.0001 *
SD	0.78	0.69	5.01	
Median	2.65	2.43	−8.09	
Min	0.86	0.78	−39.46	
Max	4.86	4.49	−0.14	
**SCA Low vs. High Brightness**				
	**Low**	**High**		
	**SCA**	**SCA**	**Modification (%)**	***p***
Average	0.87	1.10	28.71	<0.0001 *
SD	0.39	0.46	17.71	
Median	0.83	1.05	26.06	
Min	0.17	0.19	1.89	
Max	2.47	2.91	90.00	
**CVI Low vs. High Brightness**				
	**Low**	**High**		
	**CVI**	**CVI**	**Modification (%)**	***p***
Average	76.09	69.98	−8.01	<0.0001 *
SD	4.49	4.45	2.82	
Median	75.52	69.33	−8.01	
Min	64.43	56.66	−17.13	
Max	86.57	83.38	−0.38	

TCA: total choroidal area, CVI: choroidal vascularity index, SCA: stromal choroidal area, LCA: luminal choroidal area, SD: standard deviation, Min: minimum; Max: maximum; *: statistically significant.

**Table 2:** TCA, LCA, SCA, CVI and their differences (in %), using the fixed tracking mode for the total choroidal area, at brightness levels 8 and 16 brightness. TCA, LCA and SCA are in mm^2^.

**TCA Low vs. High Brightness**				
	**Low**	**High**		
	**TCA**	**TCA**	**Modification (%)**	***p***
Average	3.52	3.52	0.0	0.500
SD	1.15	1.15	0.0	
Median	3.43	3.43	0.0	
Min	1.15	1.15	0.0	
Max	6.94	6.94	0.0	
**LCA Low vs. High Brightness**				
	**Low**	**High**		
	**LCA**	**LCA**	**Modification (%)**	***p***
Average	2.64	2.43	−7.73	<0.0001 *
SD	0.78	0.72	2.82	
Median	2.60	2.40	−7.91	
Min	0.90	0.89	−19.49	
Max	4.86	4.63	−0.79	
**SCA Low vs. High Brightness**				
	**Low**	**High**		
	**SCA**	**SCA**	**Modification (%)**	***p***
Average	0.88	1.08	26.32	<0.0001 *
SD	0.40	0.47	13.96	
Median	0.85	1.06	23.78	
Min	0.21	0.26	2.80	
Max	2.47	3.01	90.58	
**CVI Low vs. High Brightness**				
	**Low**	**High**		
	**CVI**	**CVI**	**Modification (%)**	***p***
Average	75.96	70.07	−7.73	<0.0001 *
SD	4.74	4.61	2.82	
Median	75.50	69.42	−7.91	
Min	64.43	56.65	−19.49	
Max	88.92	80.41	−0.79	

TCA: total choroidal area, CVI: choroidal vascularity index, SCA: stromal choroidal area, LCA: luminal choroidal area, SD: standard deviation, Min: minimum; Max: maximum; *: statistically significant.

## Data Availability

Data are unavailable due to privacy or ethical restrictions.

## Supplementary Materials

The following supporting information can be downloaded at https://www.mdpi.com/article/10.3390/jcm13041020/s1. Figure S1: comparison between luminal choroidal area (LCA) in mm^2^ at brightness levels 8 and 16, obtained by selecting the manual tracking mode of the total choroidal area (TCA); Figure S2: comparison between luminal choroidal area (LCA) in mm^2^ at brightness levels 8 and 16, obtained by selecting a fixed total choroidal area (TCA); Figure S3: comparison between stromal choroidal area (SCA) in mm^2^ at brightness levels 8 and 16, obtained by selecting the manual tracking mode of the total choroidal area (TCA); Figure S4: comparison between stromal choroidal area (SCA) in mm^2^ at brightness levels 8 and 16, obtained by selecting a fixed total choroidal area (TCA); Figure S5: comparison between CVI at brightness levels 8 and 16, obtained by selecting the total choroidal area (TCA) manual tracking mode; Figure S6: comparison between CVI at brightness levels 8 and 16, obtained by selecting a fixed total choroidal area (TCA); Figure S7: comparison between total choroidal area (TCA) in mm^2^ at brightness levels 8 and 16, obtained by selecting the TCA manual tracking mode; Figure S8: comparison between total choroidal area (TCA) in mm^2^ at brightness levels 8 and 16, obtained by selecting a fixed TCA area.
