# Supplementary material for: Impact of Brightness on Choroidal Vascularity Index
Source: J Clin Med. 2024 Feb 10;13(4):1020. doi: 10.3390/jcm13041020 (PMC10889141; doi:10.3390/jcm13041020)

Supplementary Materials

Figure S1. Comparison between luminal choroidal area (LCA) in mm[2] at 8 and 16 brightness levels, obtained selecting manual tracking mode of the total choroidal area (TCA).

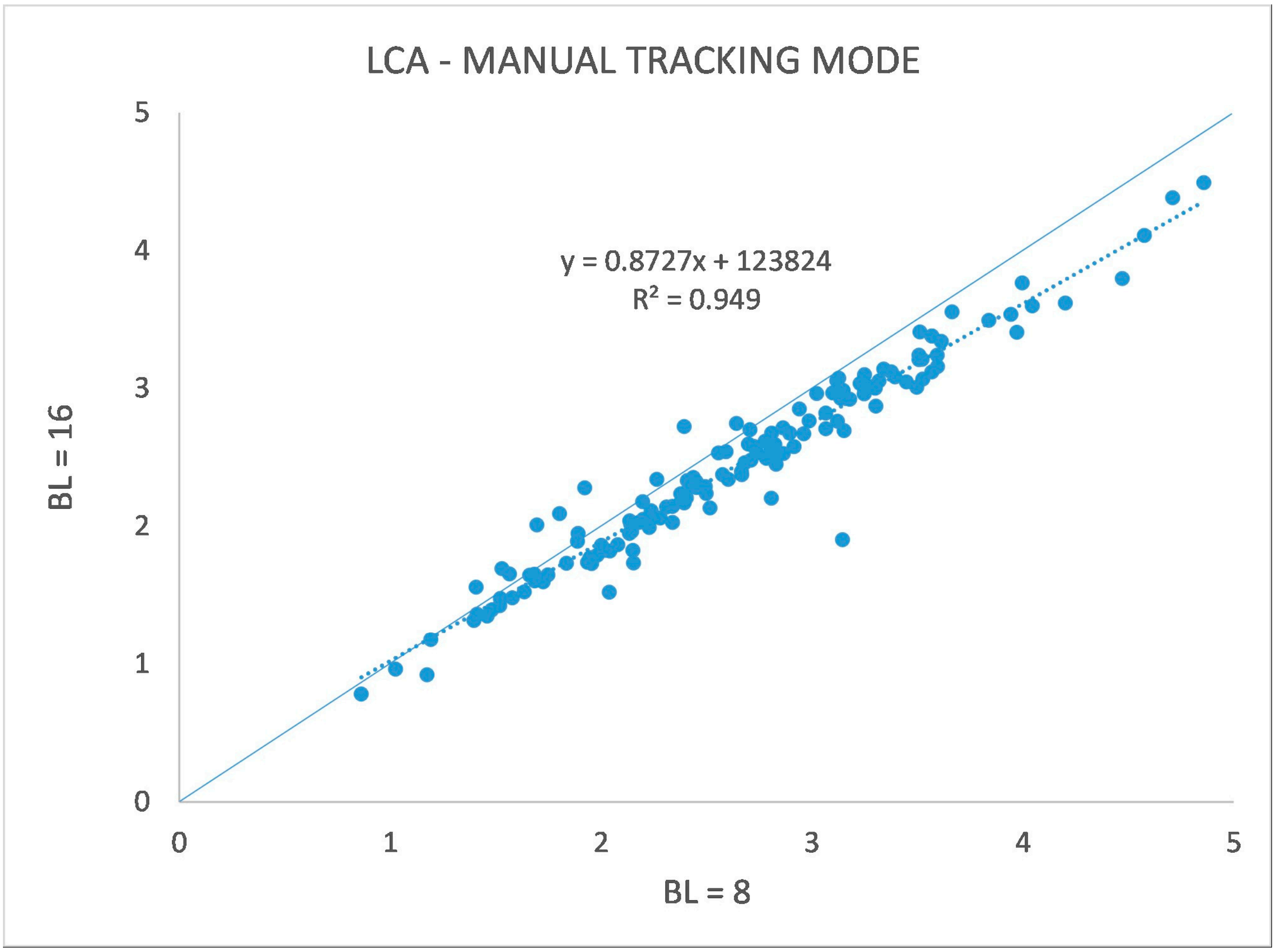

Figure S2. Comparison between luminal choroidal area (LCA) in mm^2 at 8 and 16 brightness levels, obtained selecting a fixed total choroidal area (TCA).

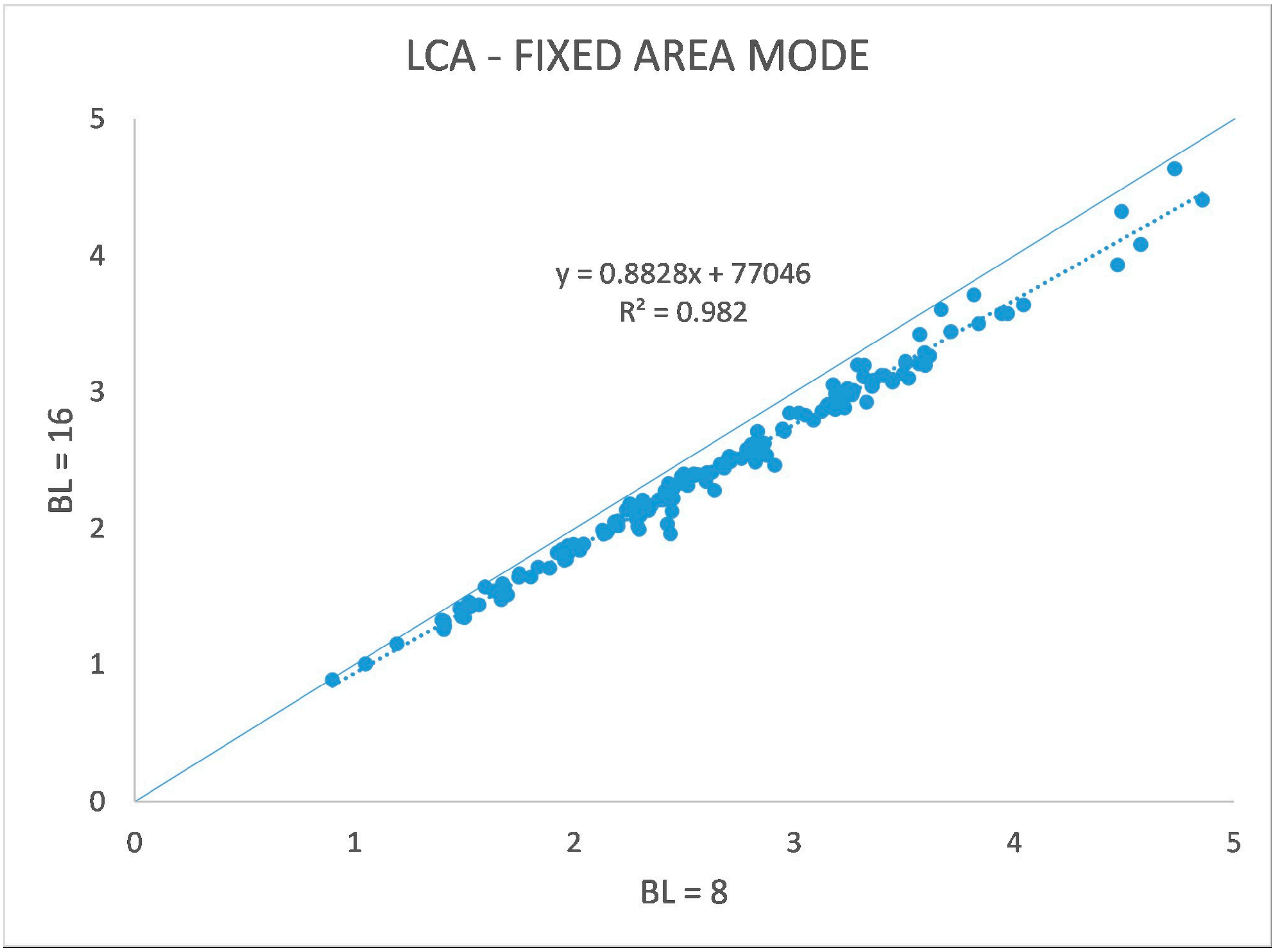

Figure S3. Comparison between stromal choroidal area (SCA) in mm² at 8 and 16 levels, obtained selecting manual tracking mode of the total choroidal area (TCA).

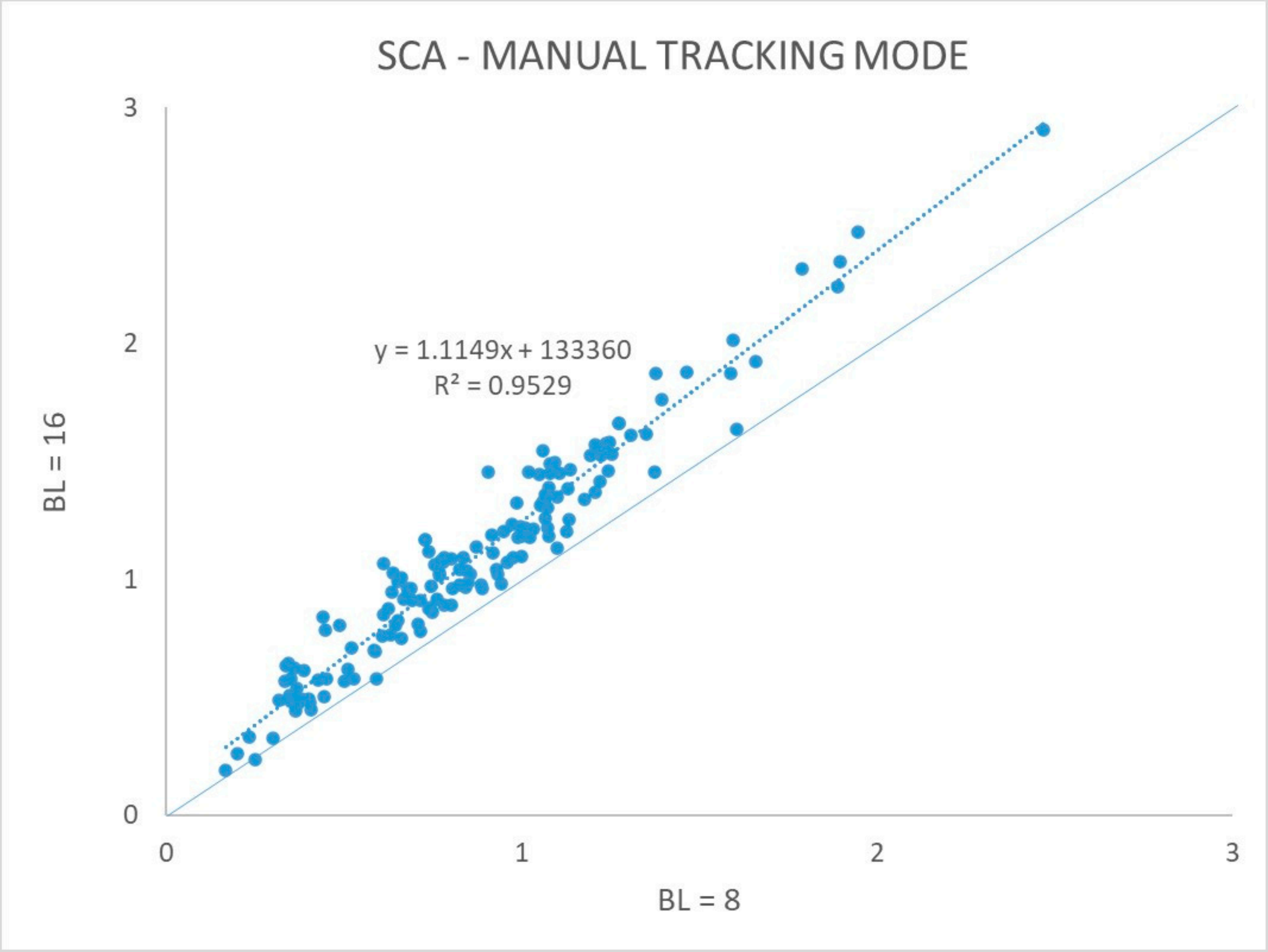

Figure S4. Comparison between stromal choroidal area (SCA) in mm² at 8 and 16 brightness levels, obtained selecting a fixed total choroidal area (TCA).

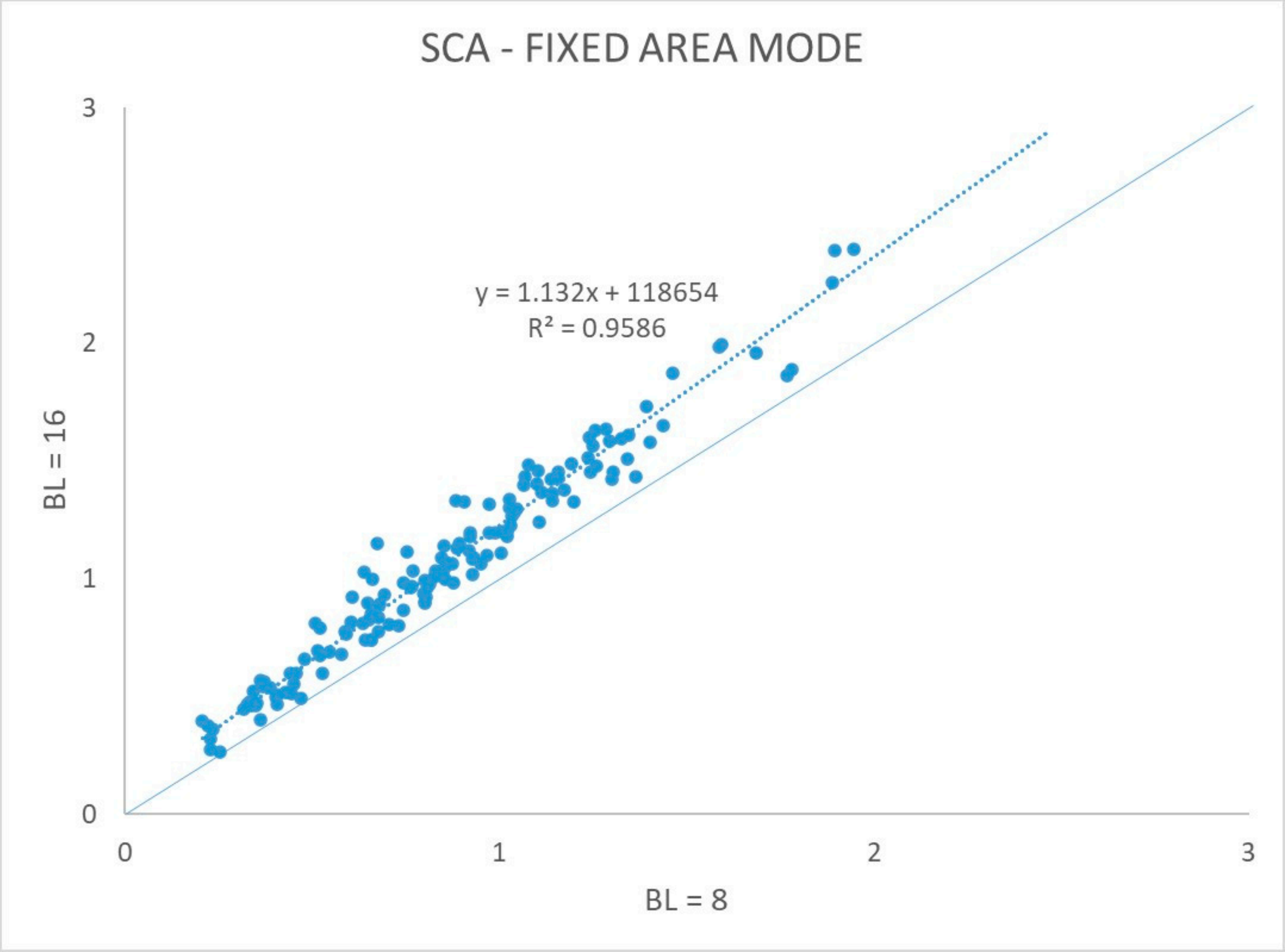

Figure S5. Comparison between CVI at 8 and 16 brightness levels, obtained selecting total choroidal area (TCA) manual tracking mode.

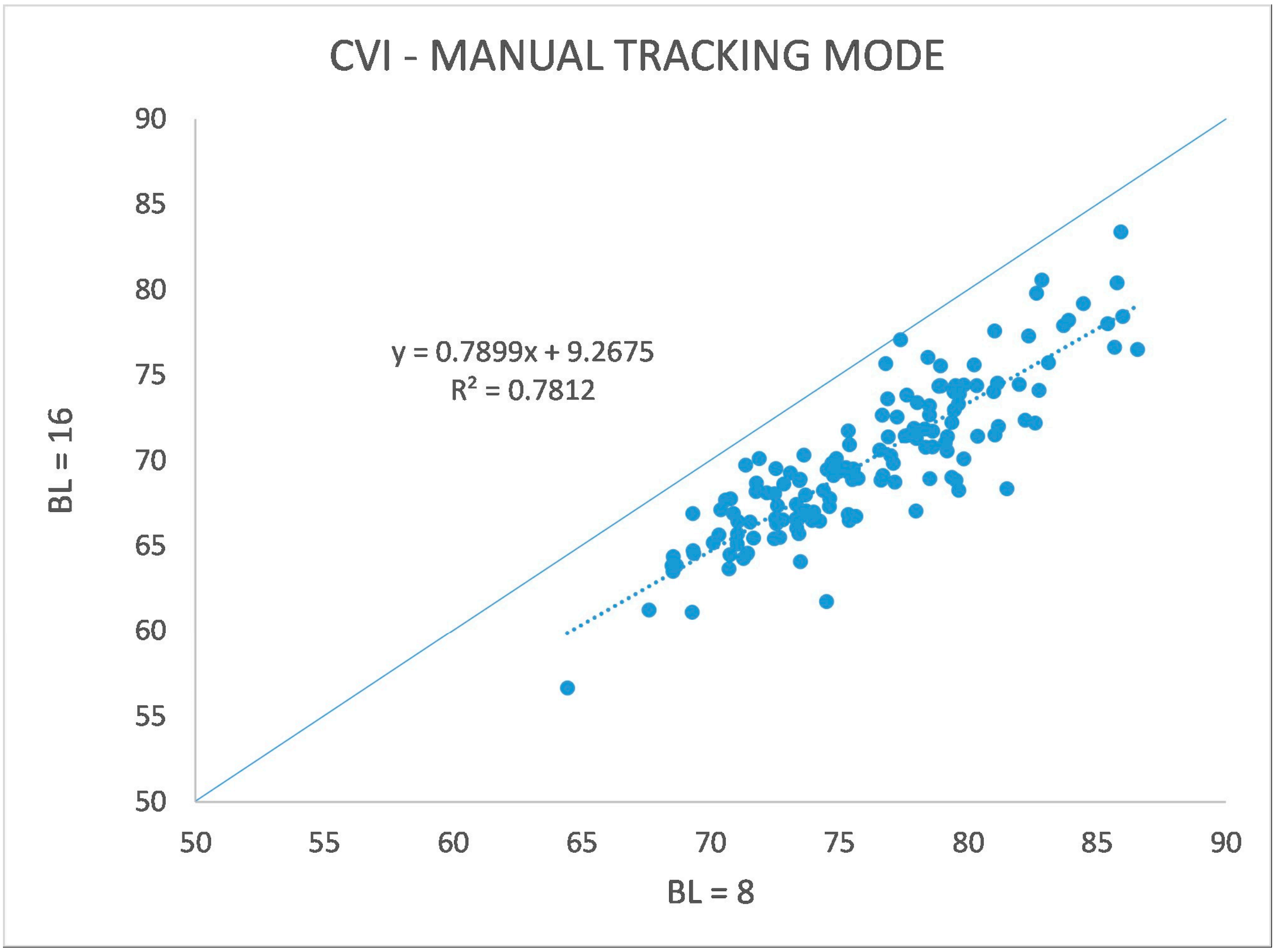

Figure S6. Comparison between CVI at 8 and 16 brightness levels, obtained selecting a fixed total choroidal area (TCA).

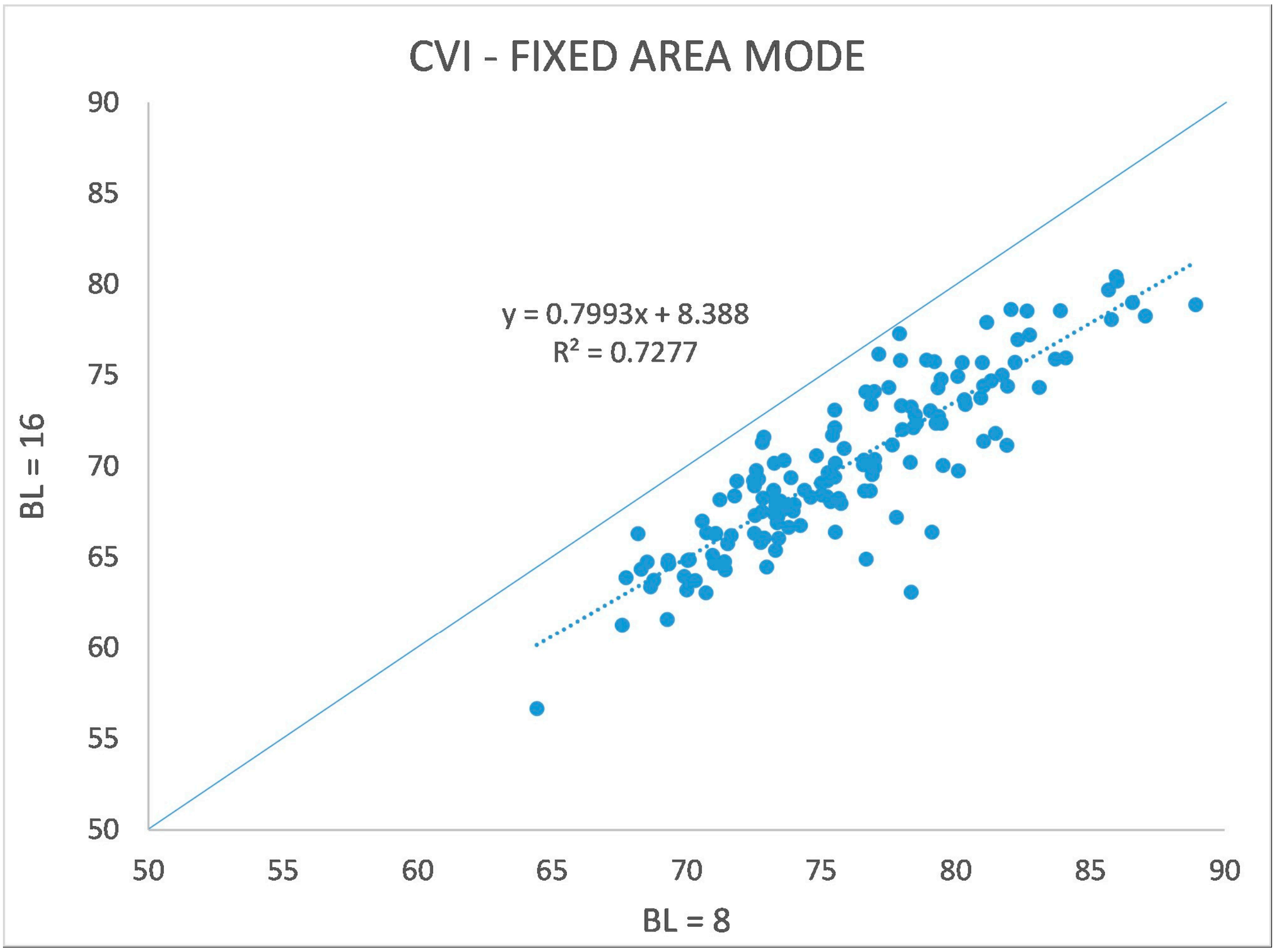

Figure S7. Comparison between total choroidal area (TCA) in mm² at 8 and 16 brightness levels, obtained selecting TCA manual tracking mode.

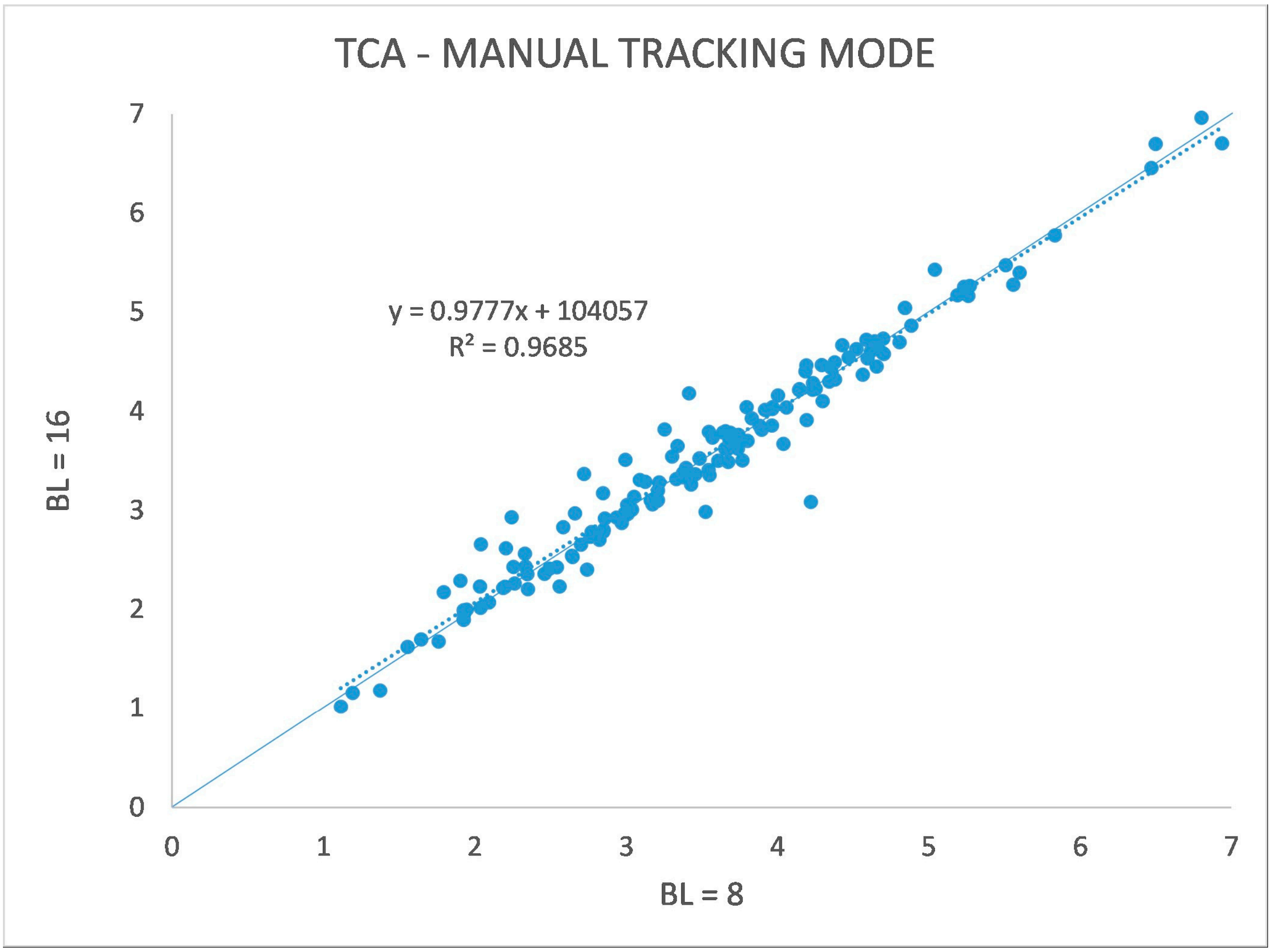

Figure S8. Comparison between total choroidal area (TCA) in mm² at 8 and 16 brightness levels, obtained selecting a fixed TCA area.

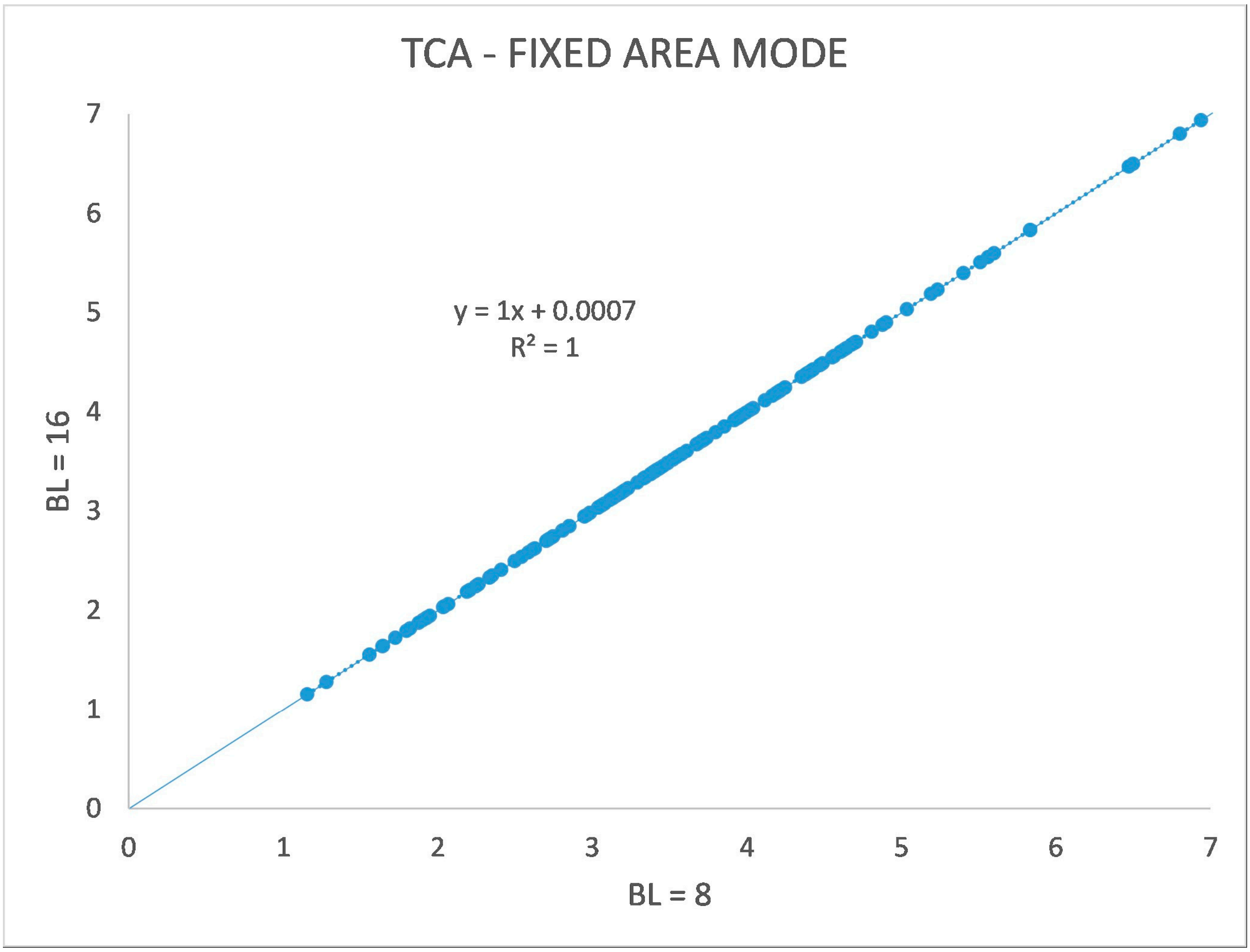

Supplement: Supplementary file 1 [file jcm-13-01020-s001.zip › jcm-2771402-supplementary.pdf]
